# Supplementary material for: Observation of an anisotropic Dirac cone reshaping and ferrimagnetic spin polarization in an organic conductor
Source: Nat Commun. 2016 Aug 31;7:12666. doi: 10.1038/ncomms12666 (PMC5013692; doi:10.1038/ncomms12666)
Supplement: Supplementary Information — Supplementary Figures 1-14, Supplementary Discussion and Supplementary References [file ncomms12666-s1.pdf]

Supplementary Information

Supplementary Figures

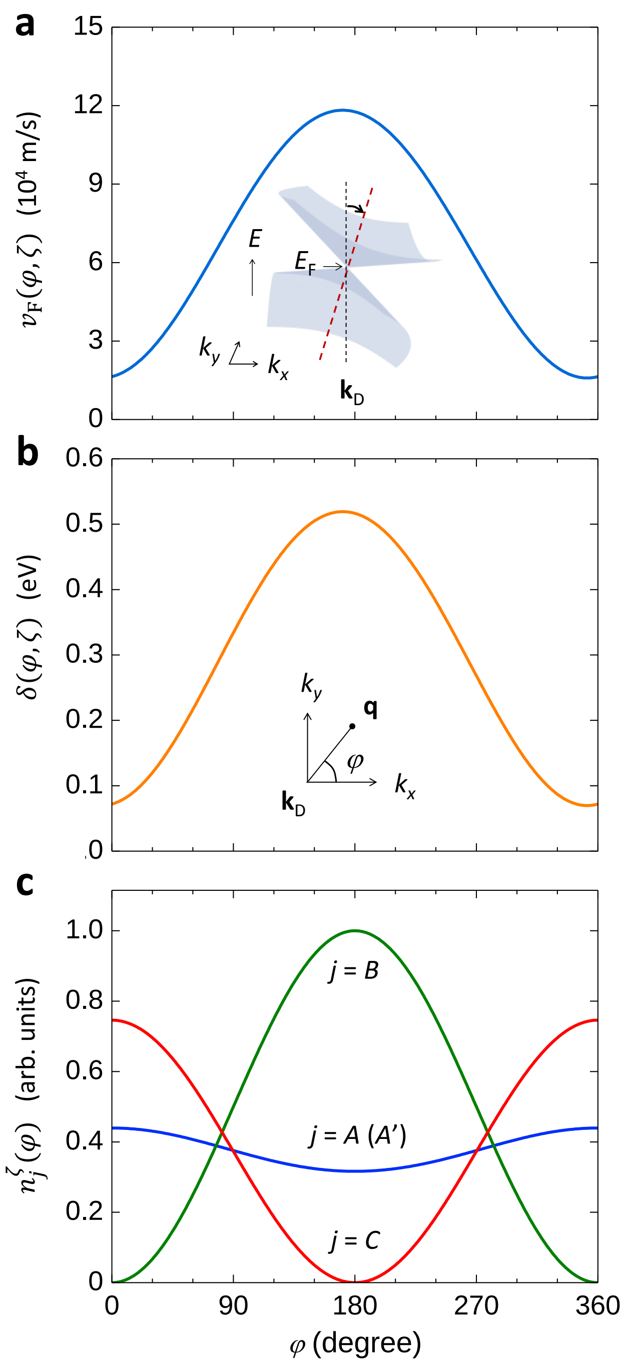

Supplementary Fig. 1 | Anisotropic Fermi velocity, energy cutoff and site-spectral weight. (**a-c**) The angular dependence of the Fermi velocity $v_F(\varphi,\zeta)$ (**a**), the energy

cut-off in the renormalization-group (RG) calculation $\delta(\varphi,\zeta) = \hbar v_F(\varphi,\zeta)\Lambda$ (**b**) and the site-spectral weight $n_j^{\zeta}(\varphi)$ for the non-equivalent site $j = A$ (= A') (blue), B (green) and C (red) (**c**) in the unit cell of α-(BEDT-TTF)$_2$I$_3$ (α-I$_3$) plotted as a function of the angle φ around the Dirac point (at $\mathbf{k}_D$). Here , φ is measured from the k_x-axis in the 2D **k**-space, $\mathbf{q} = \mathbf{k} - \mathbf{k}_D = q(\cos\varphi\,, \sin\varphi)$ [inset of (**b**)], Λ is a momentum cut-off and $\zeta = \pm$ is the band index distinguishing the conduction band ($\zeta = +$) and the valance band ($\zeta = -$). The curves in the figure are calculated for the conduction band (ζ = +) from Eqs. 11-13 based on the effective tight-binding model for α-I$_3$ in Ref. (*1*). For (**b**), the momentum cut-off of Λ = 0.667 Å^{-1} is introduced, which is circular around the Dirac point and corresponds to the size of the inverse lattice constant (see **Methods**). In (**c**), the amplitude of $n_j^{\zeta}(\varphi)$ is normalized to the maximum of $n_j^{\zeta}(\varphi)$ at the site $j = B$, in accordance with Fig. 1, d-f. Inset of (**a**): the schematic illustration of the tilted Dirac cone in α-I$_3$ around the Dirac point at $\mathbf{k}_D$, where the Fermi energy E_F is fixed at the band-crossing point due to the stoichiometry. The cone is tilted toward the k_x-axis, which causes the large anisotropy of $v_F(\varphi,\zeta)$. Note that all curves shown here have an exactly opposite phase relation for the valence band ($\zeta = -$) (*1*): $v_F(\varphi,\zeta = +) = v_F(\varphi + \pi, \zeta = -)$, $\delta(\varphi,\zeta = +) = \delta(\varphi + \pi, \zeta = -)$ and $n_j^{\zeta=+}(\varphi) = n_j^{\zeta=-}(\varphi + \pi)$.

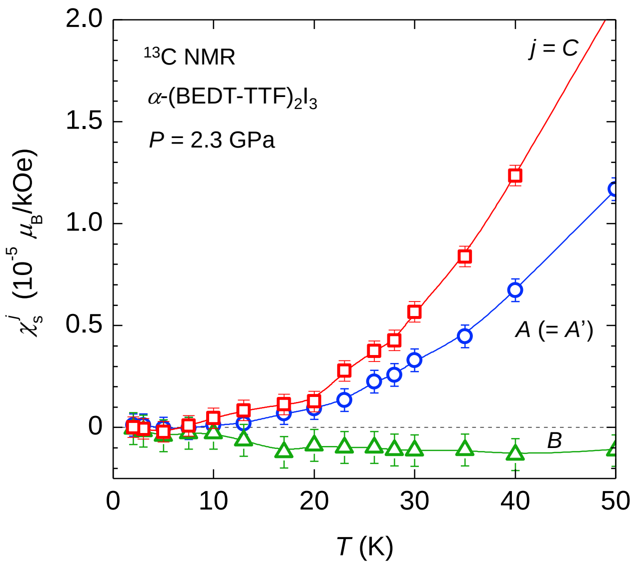

Supplementary Fig. 2 | Close up of the low-temperature part of the electron-spin susceptibility. Temperature, T, dependence of the local electron-spin susceptibility $\chi_s^j(T)$ in α-I_3 (P = 2.3 GPa) for the non-equivalent site $j = A$ (= A') (circles), B (triangles) and C (squares) in the unit cell, which is the low-T close up of Fig. 2d. The susceptibilities are determined from the ^{13}C-NMR spectra at H = 6 T applied in the crystalline ab-plane at the field orientation $\psi = 60^{\circ}$ for the site A (= A') and at $\psi = 120^{\circ}$ for the site B and C, where ψ is the angle of the magnetic field in the ab-plane measured from the a-axis (Fig. 2c). Error bars are the standard deviation of the size $\sim 10^{-6}$ μ_B kOe^{-1}. Lines stand for the guide to the eyes.

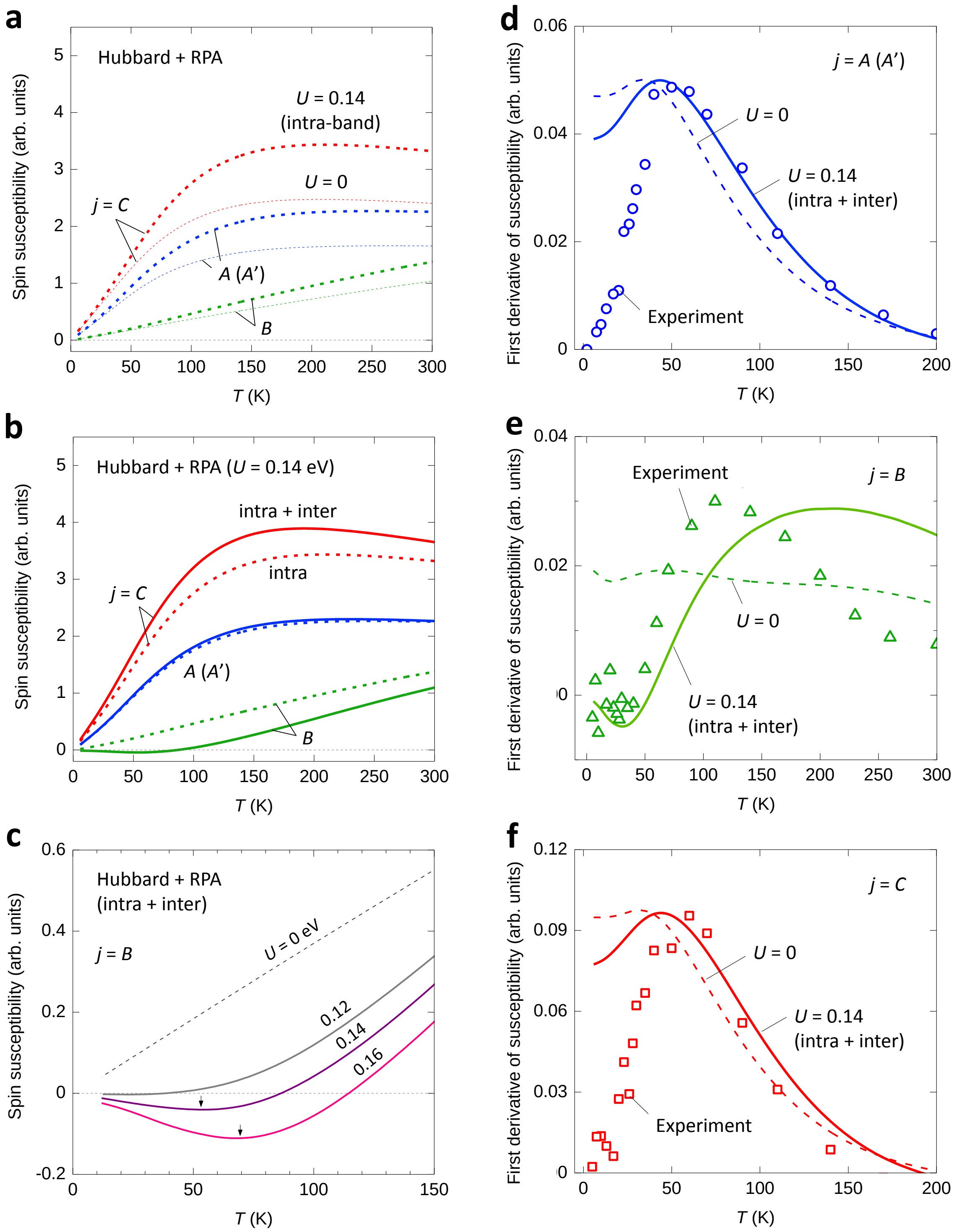

Supplementary Fig. 3 | Simulations based on the random phase approximation. (**a** and **b**) Calculated temperature, *T*, dependence of the local-electron spin susceptibility in α-I_3 for the non-equivalent site *j* = *A* (= *A*'), *B* and *C* in the unit cell in terms of the

Hubbard model (Eq. 20) and the hopping integrals of Eqs. 6, 7, 21 and 22 within the random phase approximation (RPA) (see **Methods** for details). In (**a**), the T dependence of the intra-band component of the RPA susceptibility, $\chi_{\mathrm{RPA}}^{j,\ \mathrm{intra}}$ (Eq. 30), is plotted for a finite on-site Hubbard interaction (U = 0.14 eV; thick dashed curves) together with the non-interacting case (U = 0; dotted curves). In (**b**), the total RPA susceptibility, χ_{RPA}^{j} (Eq. 29), is plotted as a function of T (intra-band + inter-band; solid curves) in comparison to the intra-band component $\chi_{\mathrm{RPA}}^{j,\ \mathrm{intra}}$ using U = 0.14 eV (dashed curves). (**c**) The calculated T dependence of the total RPA susceptibility on the site $j = B$, χ_{RPA}^{B}, plotted for different values of the Hubbard interaction U. A negative susceptibility ($\chi_{\mathrm{RPA}}^{B} < 0$) with a negative slope ($d\chi_{\mathrm{RPA}}^{B}/dT < 0$) appears at low temperature for $U > U_{\mathrm{C}} \approx 0.12$ eV, with the minimum position (indicated by arrows) shifted towards higher T with increasing U. (**d**-**f**) The first derivative of the calculated spin susceptibility plotted as a function of T for the non-equivalent site $j = A$ (= A') (**d**), B (**e**) and C (**f**) in the unit cell. The results for $d\chi_{\mathrm{RPA}}^{j}/dT$ (U = 0) (dashed curves), $d\chi_{\mathrm{RPA}}^{j}/dT$ (U = 0.14 eV) (solid curves) are shown together with the experimental data at 2.3 GPa (replotted from Fig. 2e).

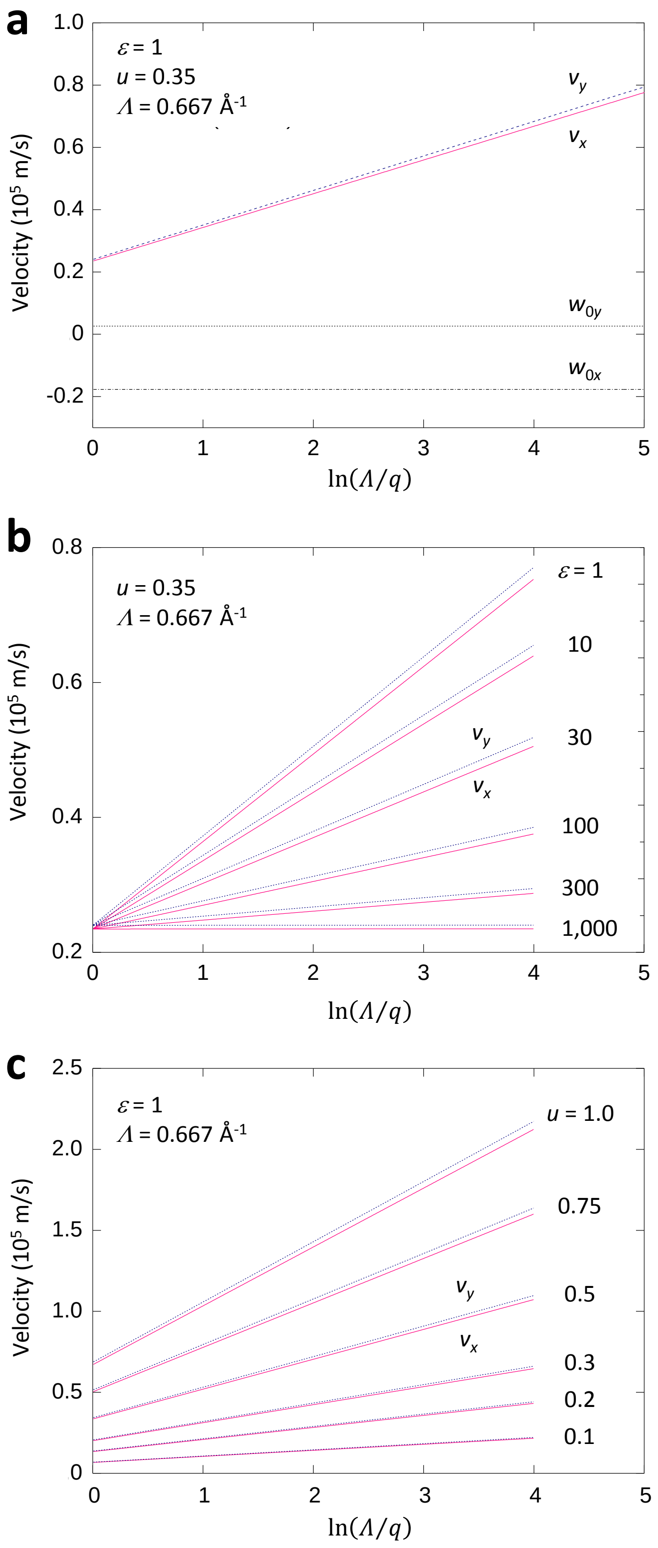

Supplementary Fig. 4 | Renormalization-group flow of velocities. (**a-c**) Calculated profiles of the renormalization-group (RG) flow of the velocities $\mathbf{w}_0 = (w_{0x}, w_{0y})$ and

$\mathbf{v} = (v_x, v_y)$ in the generalized Weyl Hamiltonian (Eq. 8), plotted as a function of $\ln(\Lambda/q)$, where $\ln x$ stands for the natural logarithm of x to the base of e, Λ = 0.667 Å^{-1} is the momentum cut-off in the 2D **k**-space which is circular around the Dirac point at $\mathbf{k}_\mathrm{D}$ (of the size of the inverse lattice constant in the crystalline *ab*-plane of α-I_3) and $q = |\mathbf{q}|$ is the wave vector measured from $\mathbf{k}_\mathrm{D}$: $\mathbf{q} = \mathbf{k} - \mathbf{k}_\mathrm{D}$. The curves are derived from the RG equation shown in Eq. 2. For the initial velocities at the cut-off momentum $q = \Lambda$, we assumed the values of $\mathbf{w}_0' = u\mathbf{w}_0^\mathrm{TB}$ and $\mathbf{v}' = u\mathbf{v}^\mathrm{TB}$ $(u \leq 1)$ (Eq. 18), where $\mathbf{w}_0^\mathrm{TB}$ and $\mathbf{v}^\mathrm{TB}$ (Eq. 12) are the velocities given by the effective TB model for α-I_3 (*1*) and u is a phenomenological parameter which incorporates the bandwidth reduction effect due to the short-range electron correction (*2*) (for details, see **Methods**). The RG flow of the velocities for the optimal parameters, (u, ε) = (0.35, 1), are shown in (**a**), which is determined from the variance fit analyses (Supplementary Fig. 7a), where ε stands for the dielectric constant. In the one-loop order of the RG calculation, $\mathbf{w}_0$ does not flow and stays constant (*3*). The ε dependence of **v** (for u = 0.35) is depicted for the parameter range ε = 1–1,000 in (**b**), while the u dependence of **v** (for ε = 1) is shown in (**c**) for the range u = 0.1–1.0.

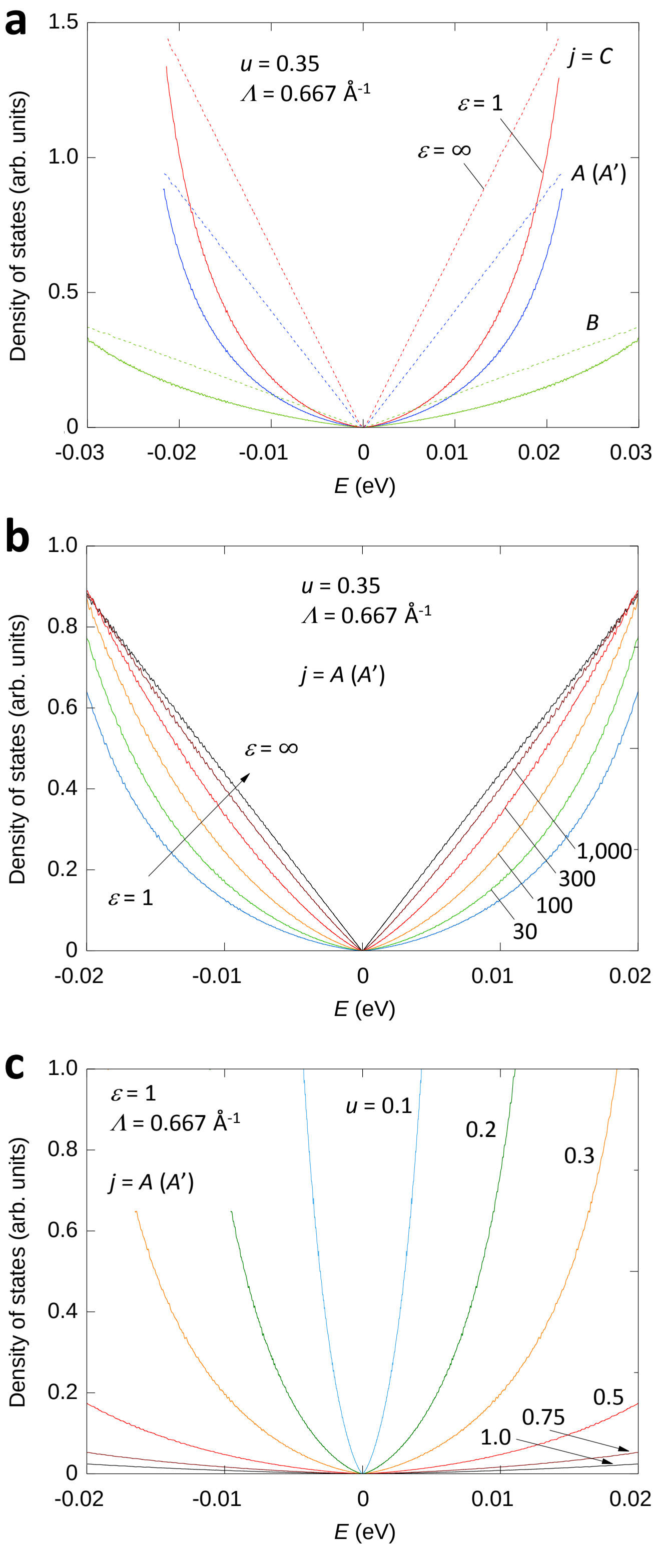

Supplementary Fig. 5 | Renormalization-group flow of the density of states. (**a-c**) The shapes of the density of states (DOS) near the Fermi energy $E = E_F$ (= 0) in α-I_3

calculated within the RG approach by means of Eqs. 2, 11, 15 and 16. In the RG calculation, the momentum cut-off of Λ = 0.667 Å^{-1} is introduced in the 2D **k**-space, which is circular around the Dirac point and has the size of the inverse lattice constant in the crystalline *ab*-plane of α-I_3. For the initial velocities at the cut-off momentum, we assumed the values of $\mathbf{w}_0' = u\mathbf{w}_0^{\mathrm{TB}}$ and $\mathbf{v}' = u\mathbf{v}^{\mathrm{TB}}$ $(u \leq 1)$ (Eq. 18), where $\mathbf{w}_0^{\mathrm{TB}}$ and $\mathbf{v}^{\mathrm{TB}}$ (Eq. 12) are the velocities given by the effective TB model for α-I_3 (*1*) and u is a phenomenological parameter which incorporates the bandwidth reduction effect due to the electron correction (*2*) (for details, see **Methods**). In (**a**), the RG corrected profiles of the DOS are presented for the non-equivalent site *j* = *A* (= *A*'), *B* and *C* in the unit cell for the optimal parameters, (u, ε) = (0.35, 1) (solid curves), and in the absence of the long-range Coulomb interaction, (u, ε) = (0.35, ∞) (dashed lines), where ε is the dielectric constant. Note that the optimal parameters are determined from the variance fit analyses in Supplementary Fig. 7a. The ε dependence of the shape of the *A*(*A*')-site DOS (for u = 0.35) is depicted in (**b**) for the parameter range ε = 1–1,000 and ε = ∞, while its u dependence (for ε = 1) is shown in (**c**) for the range u = 0.1–1.0.

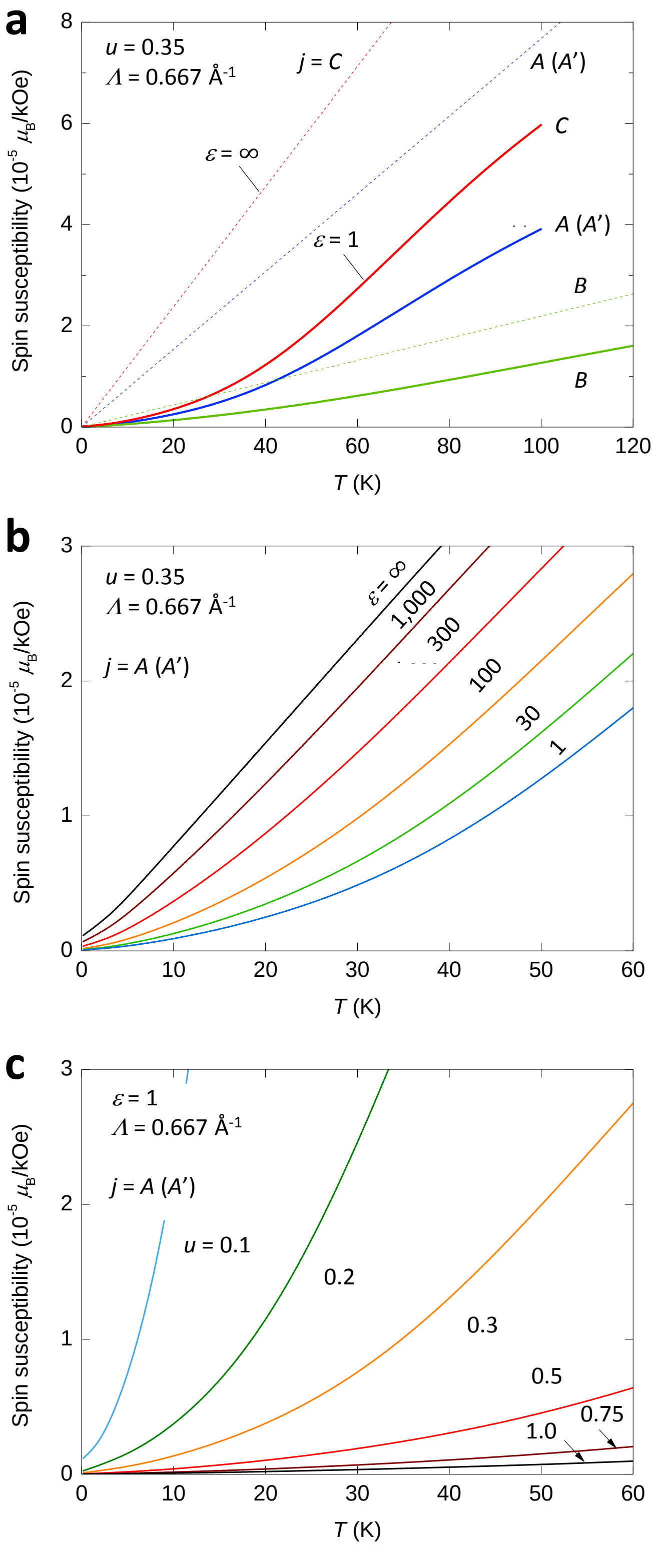

Supplementary Fig. 6 | Renormalization-group flow of electron spin susceptibilities.

(**a-c**) The temperature, T, dependence of the local-electron spin susceptibility in α-I_3

calculated within the RG approach by means of Eqs. 2, 11, 15 and 17. In the RG calculation, the momentum cut-off of Λ = 0.667 Å^{-1} is introduced in the 2D **k**-space, which is circular around the Dirac point and has the size of the inverse lattice constant in the crystalline *ab*-plane of α-I_3. For the initial velocities at the cut-off momentum, we assumed the values of $\mathbf{w}_0' = u\mathbf{w}_0^{\mathrm{TB}}$ and $\mathbf{v}' = u\mathbf{v}^{\mathrm{TB}}$ $(u \leq 1)$ (Eq. 18), where $\mathbf{w}_0^{\mathrm{TB}}$ and $\mathbf{v}^{\mathrm{TB}}$ (Eq. 12) are the velocities given by the effective TB model for α-I_3 (*1*) and u is a phenomenological parameter associated with the bandwidth reduction effect due to the electron correction (*2*) (for details, see **Methods**). In (**a**), the calculated RG flow of the electron spin susceptibility are presented for the non-equivalent site *j* = *A* (= *A*'), *B* and *C* in the unit cell for the optimal parameters, (u, ε) = (0.35, 1) (solid curves), and in the absence of the long-range Coulomb interaction, (u, ε) = (0.35, ∞) (dashed lines), where the optimal values of (u, ε) are determined from the variance fit analyses in Supplementary Fig. 7a (ε: the dielectric constant). The ε dependence of the profiles of the *A*(*A*')-site susceptibility (for u = 0.35) is depicted in (**b**) for the parameter range ε = 1–1,000 and ε = ∞, while its u dependence (for ε = 1) is shown in (**c**) for the range u = 0.1–1.0. Note that the low-*T* levelling off of the susceptibility, for large ε or small u in (**b**) and (**c**), is due to the finite DOS at E_F induced by the electron Zeeman effect, while this effect is neglected in (**a**) (for ε = ∞) by taking a low-field limit for the sake of simplicity.

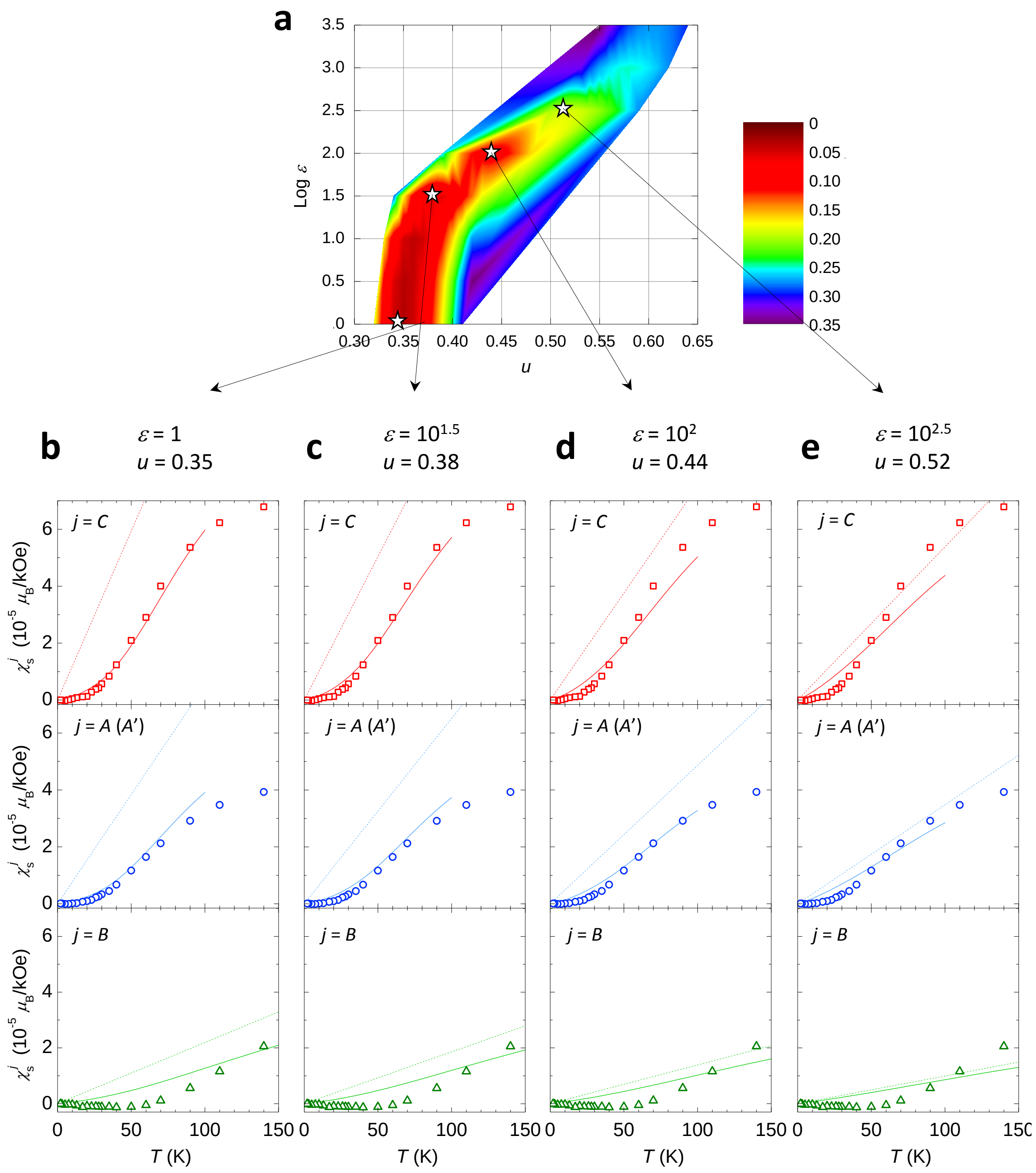

Supplementary Fig. 7 | Fitting analyses of electron spin susceptibilities. (**a**) The calculated variance, $\mathrm{Var}(\chi_s)$ (Eq. 19), on the basis of the RG approach is plotted as a function of u and ε, where the label bar indicates the magnitude of $\mathrm{Var}(\chi_s)$, stars represent characteristic points around the variance minimum, u is a phenomenological fitting parameter reflecting the bandwidth reduction (see below), ε is the dielectric constant and Log x stands for the logarithm of x to the base of 10. Note that the fitting is

carried out using the experimental local-electron spin susceptibility in α-I_3 (P = 2.3 GPa), $\chi_s^j(T)$ (Fig. 2d), for the non-equivalent site j = A (= A') and C in the unit cell. In the RG calculation, the momentum cut-off of Λ = 0.667 Å^{-1} is introduced in the 2D **k**-space, which is circular around the Dirac point and has the size of the inverse lattice constant in the crystalline *ab*-plane of α-I_3. For the initial velocities at the cut-off momentum, we assumed the values of $\mathbf{w}_0' = u\mathbf{w}_0^{\mathrm{TB}}$ and $\mathbf{v}' = u\mathbf{v}^{\mathrm{TB}}$ $(u \leq 1)$ (Eq. 18), where $\mathbf{w}_0^{\mathrm{TB}}$ and $\mathbf{v}^{\mathrm{TB}}$ (Eq. 12) are the velocities given by the effective TB model for α-I_3 (*1*) and u is a phenomenological parameter reflecting the bandwidth reduction effect due to the electron correction (*2*) (for details, see **Methods**). (**b**-**e**) The calculated temperature, T, dependence of the susceptibility based on Eqs. 2, 11, 15 and 17 (solid curves) for the parameters (u, ε) = (0.35, 1) (**b**), (0.38, $10^{1.5}$) (**c**), (0.44, 10^2) (**d**) and (0.52, $10^{2.5}$) (**e**) plotted together with the experimental data for the non-equivalent site j = A (= A'), B and C in the unit cell (symbols; replotted from Fig. 2d). Dotted lines indicate the T dependence for the corresponding value of u in the absence of the long-range Coulomb interaction ($\varepsilon = \infty$).

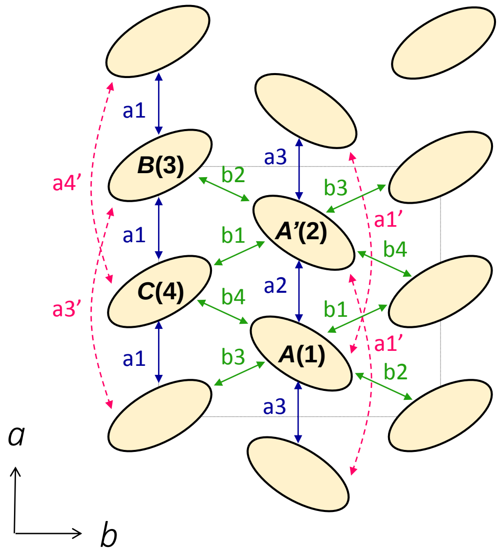

Supplementary Fig. 8 | Hopping amplitudes in the conducting layer. Schematic illustration of the anisotropic network of the transfer integrals in α-I_3 in the crystalline *ab*-plane (Fig. 1, a and b). Ellipses represent the molecular site *A*(1), *A*'(2), *B*(3) and *C*(4) in the 2D unit cell (indicated by the box). Note that *A*(1) and *A*'(2) are connected by the inversion operation in the conducting phase such that there are three non-equivalent sites in the unit cell, namely, *j* = *A* (= *A*'), *B* and *C*. The indices a1, a2, a3, b1, b2, b3 and b4 stand for the nearest-neighbour hopping, whereas a1', a3' and a4' refer to the largest next-nearest-neighbour hopping, following the definition in Ref. (*1*).

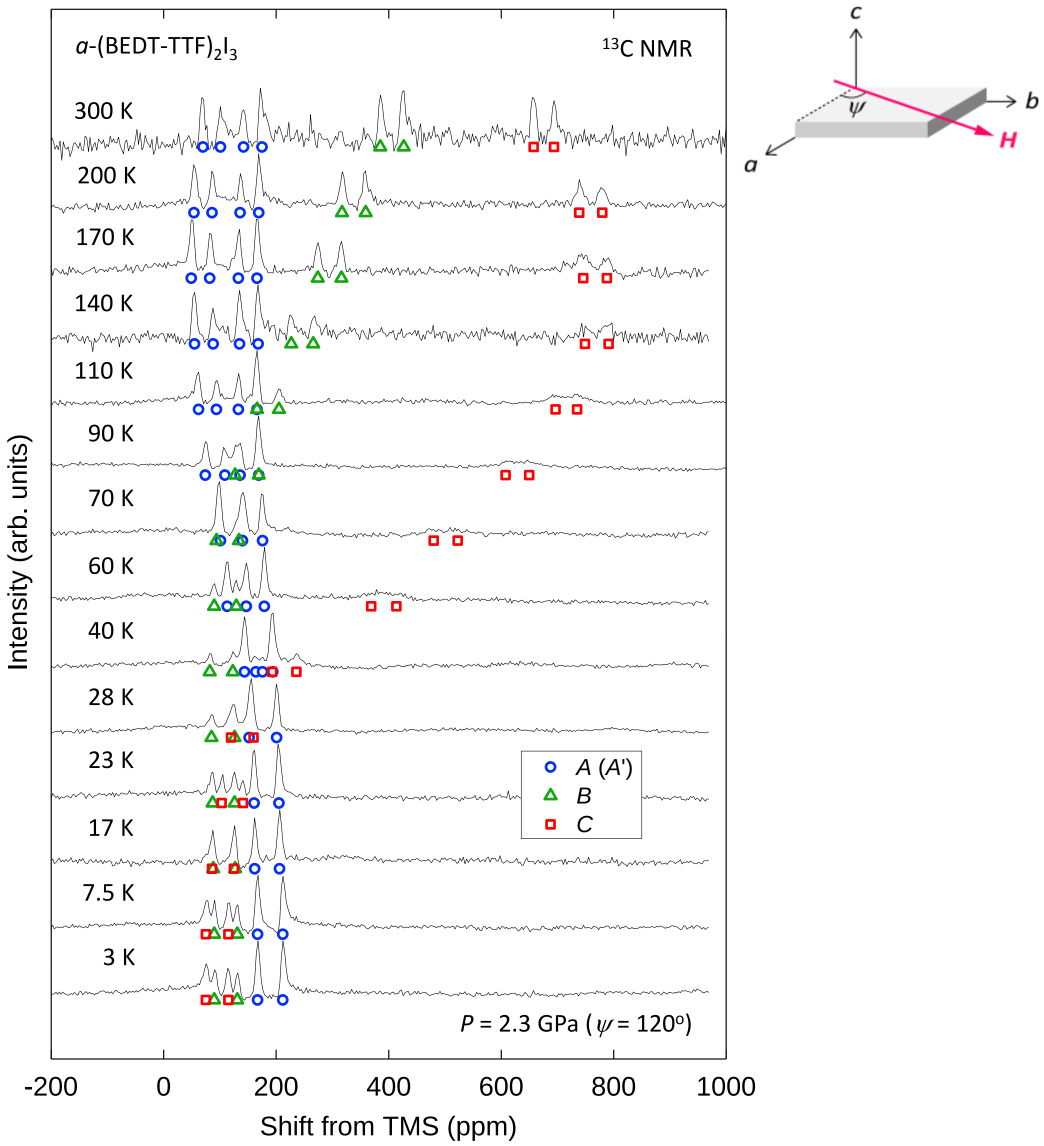

Supplementary Fig. 9 | Temperature dependence of the ^{13}C-NMR spectra. NMR spectra in α-I3 at P = 2.3 GPa are shown as a function of temperature. The magnetic field of H = 6 T is applied in the crystalline *ab*-plane in the direction $\psi = 120^{\circ}$, where ψ is measured from the *a*-axis (see the inset). The symbols denote the ^{13}C lines corresponding to the non-equivalent site $j = A$ (= *A*') (circles), *B* (triangles) and *C* (squares) in the unit cell. The temperature dependence of the local-electron spin susceptibility χ_s^j, shown in Fig. 2d, is deduced from these spectra for the site $j = B$ and *C*, while it is determined from Fig. 2a (the spectra measured at $\psi = 60^{\circ}$) for the site $j = A$ (= *A*') (for details, see **Methods**).

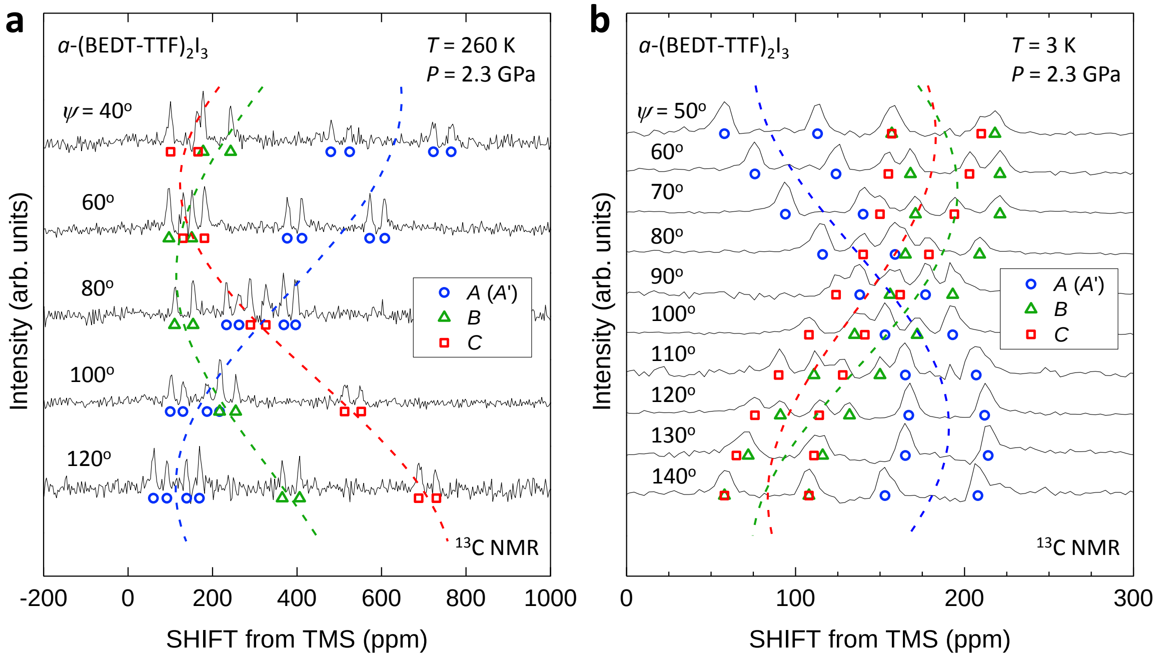

Supplementary Fig. 10 | Field-orientation dependence of the ^{13}C-NMR spectra. (**a** and **b**) Field angular dependence of the NMR spectra in α-I_3 at T = 260 K (**a**) and 3 K (**b**) (P = 2.3 GPa). The magnetic field H (= 6 T) is rotated in the conducting *ab*-plane. The field angle ψ is measured from the *a*-axis. The symbols indicate the ^{13}C lines corresponding to the non-equivalent site j = A (= A') (circles), B (triangles) and C (squares) in the unit cell. The dashed curves represent the sinusoidal fits to the angular dependence of the centre-of-gravity line shift, associated to the three non-equivalent sites (for 260 K, the fitted curves are plotted in Fig. 2b).

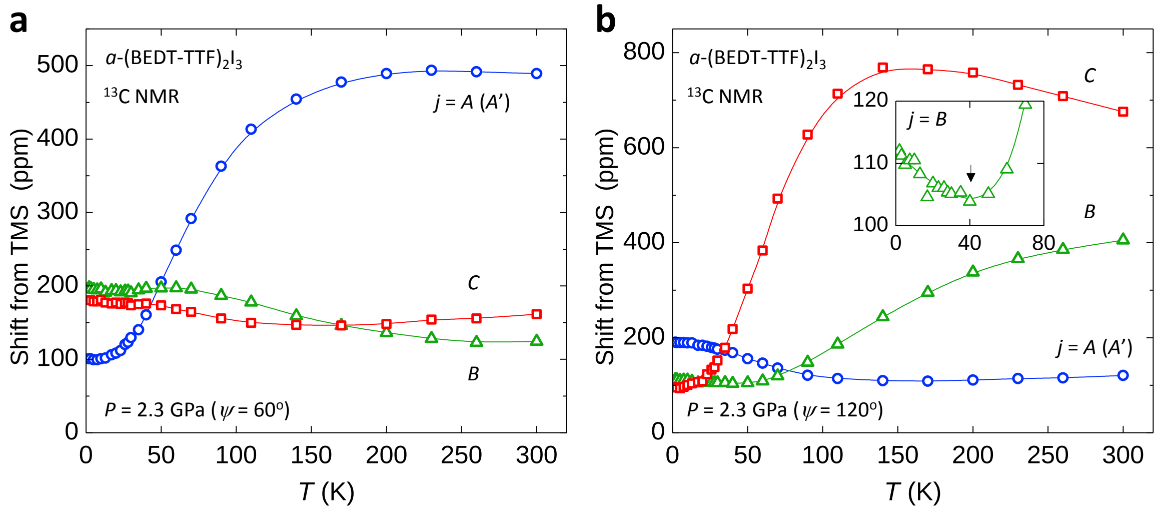

Supplementary Fig. 11 | Temperature dependence of the ^{13}C-NMR shift. (**a** and **b**) Temperature, *T*, dependence of the NMR shift in α-I_3 (*P* = 2.3 GPa) for the non-equivalent site *j* = *A* (= *A*') (circles), *B* (triangles) and *C* (squares) in the unit cell. The data are deduced from Fig. 2a for the field angle $\psi = 60^o$ (**a**) and from Supplementary Fig. 9 for $\psi = 120^o$ (**b**). The curves are the guide to the eyes. Distinct temperature dependence is observed for different field orientations at the non-equivalent sites, which reflects the difference in the sign of the hyperfine-coupling constant for different field orientations. The prominent variation of the shift on cooling originates from the strong decrease of the Knight (spin) shift as $T \to 0$ (see **Supplementary Discussion** for details). The low *T* offsets of the shift correspond to the *T*-independent chemical shift due to the core electrons. Inset of (**b**): The low-*T* close-up of the shift on the site *j* = *B* ($\psi = 120^o$). The arrow indicates the onset temperature of the low-*T* upturn (≈ 40 K), which is relevant to the ferrimagnetic spin polarization as illustrated in Fig. 6 (see the main text) (*4*).

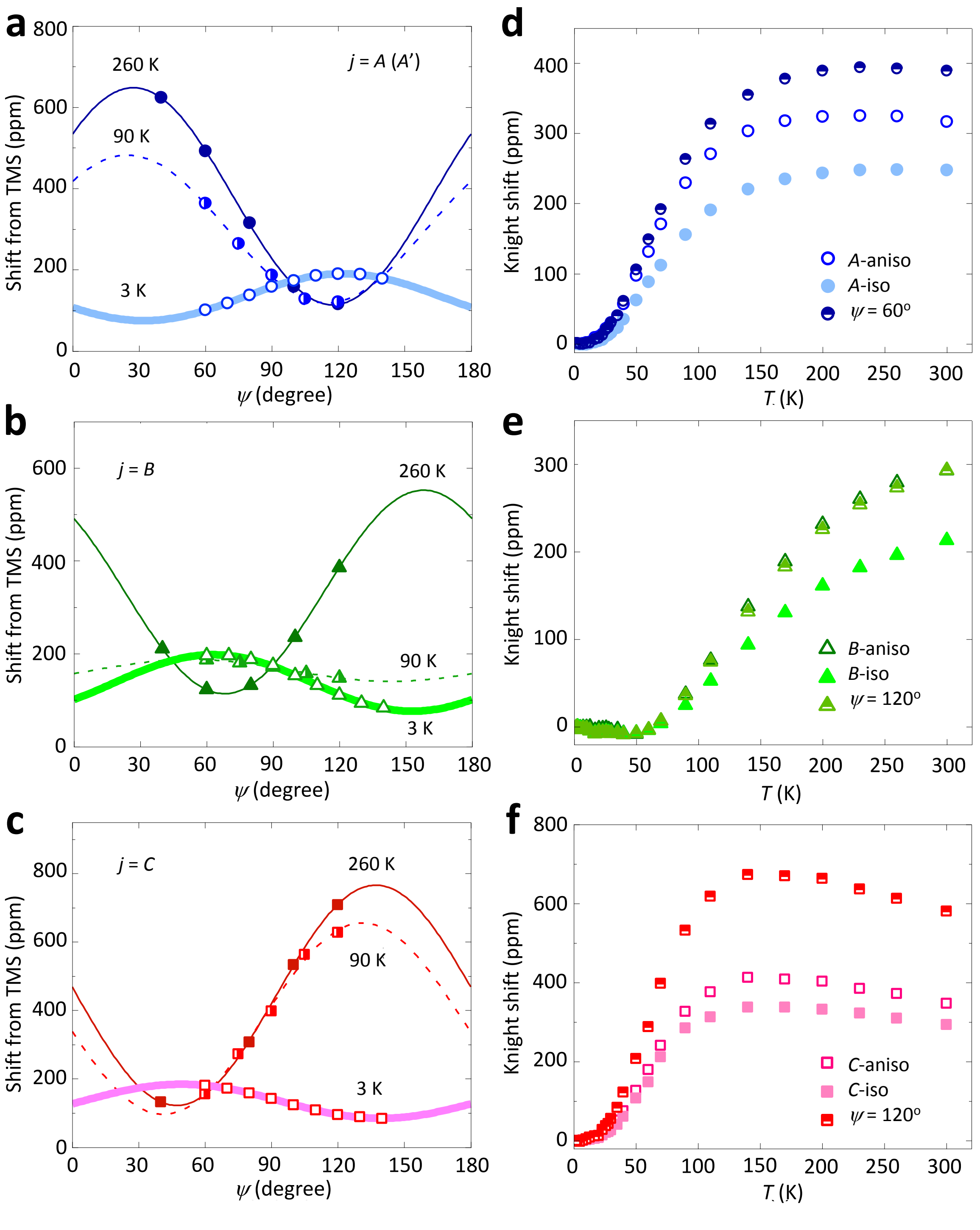

Supplementary Fig. 12 | Conversion of the ^{13}C-NMR shift to the Knight shift. (**a** to **c**) Field angular dependence of the centre-of-gravity NMR line shift in α-I_3 (P = 2.3 GPa) for the non-equivalent site j = A (= A') (**a**), B (**b**) and C (**c**) in the unit cell, at selected temperature, T, in the crystalline ab-plane. The field direction ψ is measured from the

a-axis. The solid and dashed curves represent the sinusoidal fits to the data. (**d** to **f**) The T dependence of the Knight shift for the non-equivalent site $j = A$ (= A') (**d**), B (**e**) and C (**f**) in the unit cell. For comparison, the Knight shift values corresponding to the in-plane anisotropic part, $K_{j,\,\mathrm{aniso}}^{ab}(T)$, the isotropic part, $K_j^{ab}(T)$, and a fixed orientation, $K_j(T,\psi)$, are presented, where the isotropic and anisotropic parts of the Knight shift are defined as in the Supplementary Eq. 2. The Knight shift $K_j(T,\psi)$ is converted from the total NMR shift $S_j(T,\psi)$, by subtracting the corresponding fitting result to the 3 K data, $S_j(T{=}3\ \mathrm{K},\psi)$ [thick bold curves in (**a**) to (**c**)]: $K_j(T,\psi) = S_j(T,\psi) - S_j(T = 3\ \mathrm{K},\psi)$ (see **Supplementary Discussion** for details).

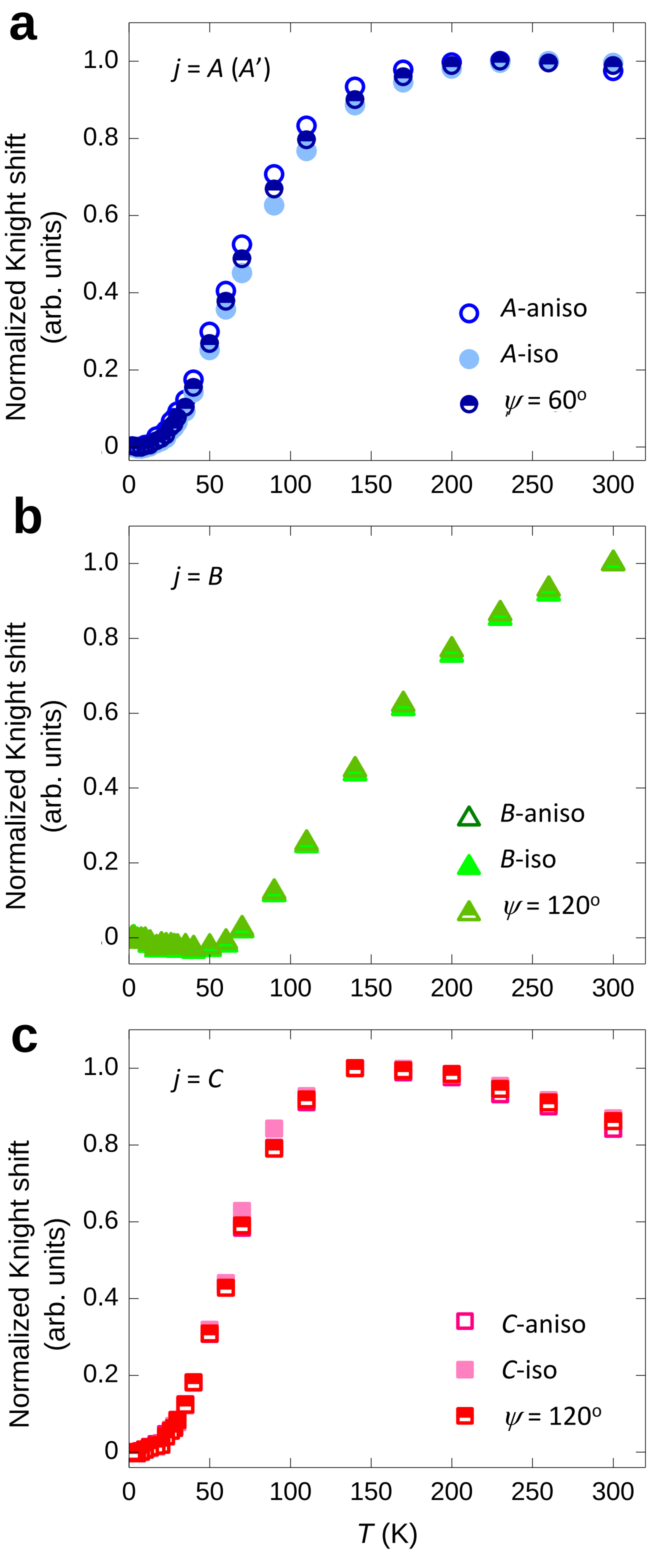

Supplementary Fig. 13 | Normalized Knight shift for different geometries. (**a** to **c**) The Knight shift in α-I_3 (P = 2.3 GPa) for the non-equivalent site $j = A$ (= A') (**a**), B (**b**)

and C (**c**) in the unit cell, plotted against temperature, T. Normalized values to the maximum are presented, which correspond to $K_{j,\,\mathrm{aniso}}^{ab}(T)$, $K_j^{ab}(T)$ and $K_j(T, \psi)$ in Supplementary Fig. 12, d to f. For all non-equivalent sites, a nice agreement is observed between three different Knight shifts, demonstrating that the hyperfine-coupling tensor in α-I_3 is T-independent (see **Supplementary Discussion** for details).

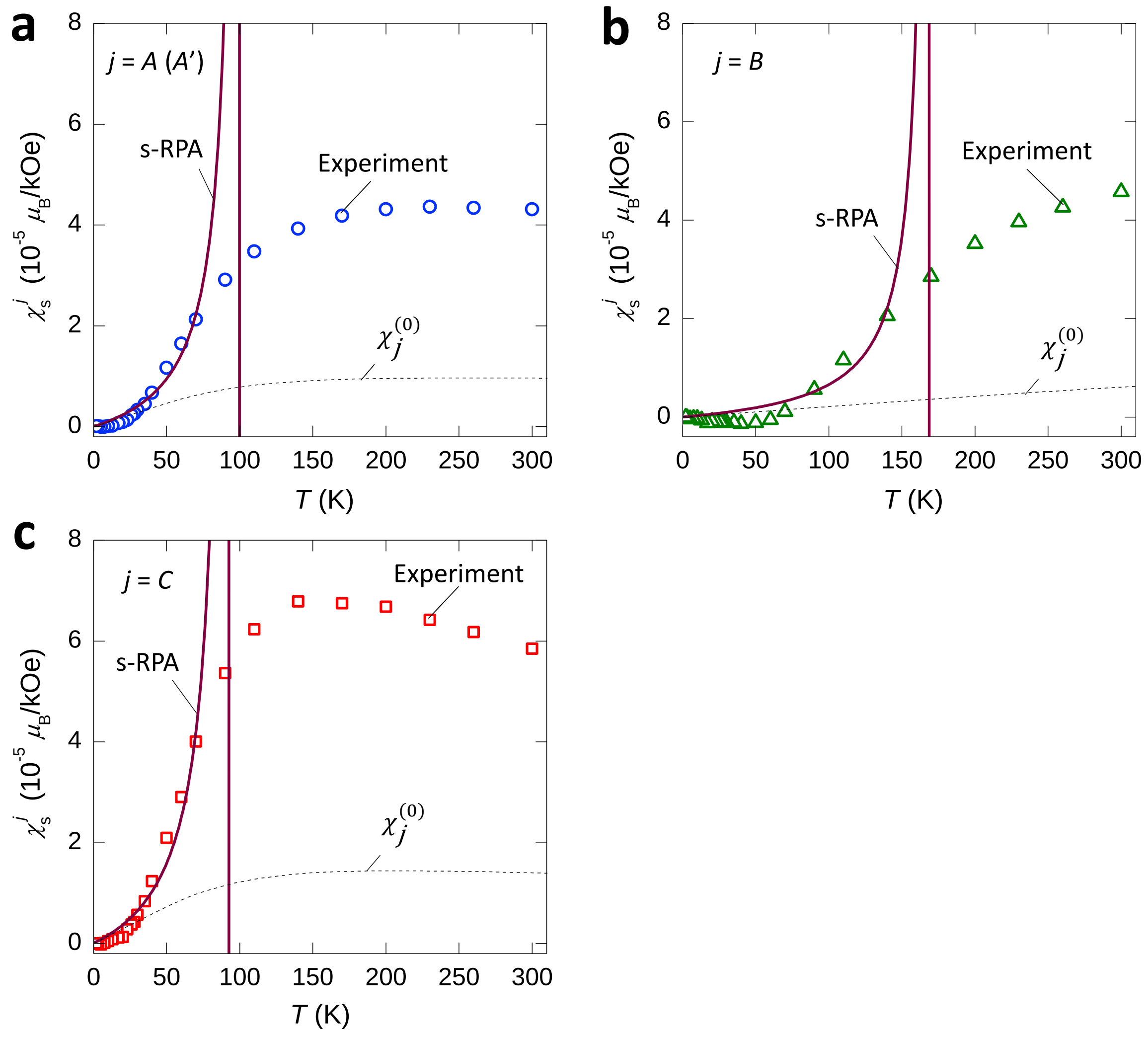

Supplementary Fig. 14 | Simplified random phase approximation fit. (**a** to **c**) The observed local-electron spin susceptibility χ_s^j in α-I_3 (P = 2.3 GPa) (symbols; replotted from Fig. 2d) are compared with the fitted curves based on the simplified random phase approximation (s-RPA), $\chi_{\text{s-RPA}}^j$ (solid curves; Eq. 32) for the non-equivalent site $j = A$ (= A') (**a**), B (**b**) and C (**c**) in the unit cell. The optimal parameters for the fitted curves are given by $U_{A(A')}$ = 0.6, U_B = 1.3 and U_C = 0.4 (in eV), respectively, where U_j is the site-dependent Hubbard interaction. The dashed curves stand for the non-interacting bare spin susceptibility $\chi_j^{(0)}$, calculated from the effective tight-binding model for α-I_3 (1). Unphysical divergence in $\chi_{\text{s-RPA}}^j$ is visible at $T \sim$ 100-170 K at all non-equivalent

sites, suggesting that the s-RPA fitting does not reproduce the experiment well (for details, see **Methods**).

Supplementary Discussion

Evaluation of the ^{13}C-Hyperfine coupling tensors under pressure in α-(BEDT-TTF)$_2I_3$. The ^{13}C-Hyperfine coupling tensors in α-(BEDT-TTF)$_2I_3$ have been reported at ambient pressure around room temperature (*5*), yet they are unknown either under pressure (P) or at low temperature (T). To estimate the P and T evolutions of the coupling tensors, we investigated the field-orientation dependence of the ^{13}C-NMR shift at various temperatures at P = 2.3 GPa. The in-plane isotropic part, K_j^{ab}, and the anisotropic part, $K_{j,\,\mathrm{aniso}}^{ab}$, of the Knight shift are evaluated on the four different sites: $j = A$, A', B and C in the unit cell (see Fig. 1, a and b). We find that their ratio, $K_j^{ab}/K_{j,\,\mathrm{aniso}}^{ab}$, does not vary as a function of T at all sites. Moreover, it is almost identical to the ratio reported at ambient pressure at T = 300 K for all sites (*5*). These results clearly indicate that there is no reason to expect particular T and P evolutions in the hyperfine coupling tensors such that one can practically use those values reported at ambient pressure (*5*) for any T and P values.

Supplementary Fig. 12, a-c, presents the field-angle (ψ) dependence of the ^{13}C-NMR shift in α-(BEDT-TTF)$_2I_3$ at P = 2.3 GPa at the non-equivalent site $j = A$ (= A'), B and C in the unit cell for selected T. The field is rotated in the conducting *ab*-plane, where the angle ψ is measured from the crystalline *a*-axis (see the inset of Supplementary Fig. 9). The shift for each site shows sinusoidal ψ dependence with a large amplitude at T = 260 K, while the amplitude decreases on cooling, and the phase almost inverts at T = 3 K. The NMR shift in BEDT-TTF-based conductors is given by a sum of the T-independent chemical (core electron) shift and the Knight (spin) shift without orbital (van-Vleck) contributions (*6*). The observed prominent T dependence in the angular profile of the total shift reflects the large T variation of the spin shift, as expected in the charge-neutral Dirac cone systems (*1,4,7*). This is because the quasiparticle excitations around the gapless point at E_F vanish at low T, and the spin shift goes to zero with decreasing temperature. Indeed, the low-T curve at T = 3 K can be well explained in terms of the chemical-shift tensor given at ambient pressure (*5,6*) and the crystal structure determined under pressure (*8*). We therefore fit to the data at T = 3 K with a sinusoidal

function (thick bold curves in Supplementary Fig. 12, a-c) and assume that the fit results provide the ψ-dependent chemical shift at this pressure value for different sites.

The observed ψ dependence of the total shift with a large amplitude at high T should be attributed to the field-orientation dependence of the Knight shift (*5,9,10*). The Knight shift, on the site j, is associated to the site-spin susceptibility, χ_s^j, in terms of the averaged hyperfine-coupling tensor, $\bar{\boldsymbol{\mathcal{A}}}_j$ (*5,9*), for the two central ^{13}C nuclei in the molecule j (inset of Fig. 1a). (We note that the two central ^{13}C nuclei are identical for the B and C molecules, while they are not for the A(A') molecule due to the inversion symmetry (*11,12*). The difference in the latter is, however, negligible as the dimerization of the molecules is weak in the α-type salts (*5,9*). The coupling tensor is, therefore, defined as an averaged quantity between the two central ^{13}C nuclei in a molecule.) At ambient pressure, the site-averaged Knight shift scales with the isotropic spin susceptibility (*5,13*). Although there are no direct reports of the susceptibility under pressure yet, it is reasonable to assume that the susceptibility is isotropic under pressure because the high-T conducting phase shows very similar properties at ambient and high pressures (*5,8-10,14-16*). The Knight shift on the site j is, then, given by the expression

$$K_j = \frac{\mathbf{H}_0 \cdot \bar{\boldsymbol{\mathcal{A}}}_j \chi_s^j \cdot \mathbf{H}_0}{|\mathbf{H}_0|^2} = \left(\bar{A}_j^{xx} {h_x}^2 + \bar{A}_j^{yy} {h_y}^2 + \bar{A}_j^{zz} {h_z}^2\right)\chi_s^j, \tag{1}$$

where $(\bar{A}_j^{xx}, \bar{A}_j^{yy}, \bar{A}_j^{zz})$ are the principal values of the tensor $\bar{\boldsymbol{\mathcal{A}}}_j$ and $\mathbf{H}_0/|\mathbf{H}_0| = \left(h_x,\ h_y,\ h_z\right)$ is the unit vector parallel to the applied magnetic field $\mathbf{H}_0$. [The principal axes are attached to the molecular frame of BEDT-TTF (*6*).] The tensor $\bar{\boldsymbol{\mathcal{A}}}_j$ is shown to be strongly anisotropic at ambient pressure (*5*). Although its value under pressure is to be determined, it is indicated that the observed angular dependence of the total NMR shift (at P = 2.3 GPa) may also reflect the anisotropy of $\bar{\boldsymbol{\mathcal{A}}}_j$.

To gain better insights into the anisotropy of $\bar{\boldsymbol{\mathcal{A}}}_j$ under pressure, the total NMR shift for the site j at the field-angle ψ, $S_j(T,\psi)$, is converted to the Knight shift, $K_j(T,\psi)$, by subtracting the corresponding fitting result to the shift data at T = 3 K: $K_j(T,\psi) = S_j(T,\psi) - S_j(T = 3\ \mathrm{K},\psi)$. Making a sinusoidal fit to the ψ dependence of $K_j(T,\psi)$ at

various T, we determine the in-plane isotropic (K_j^{ab}) and anisotropic ($K_{j,\,\mathrm{aniso}}^{ab}$) parts of the Knight shift (in the *ab*-plane) in terms of the expression

$$K_j(T,\psi) = K_j^{ab}(T) + K_{j,\,\mathrm{aniso}}^{ab}(T)\sin\{2(\psi + \phi_j)\}. \tag{2}$$

The T-independent phase ϕ_j, which is solely determined by the molecular arrangement in the 2D unit cell, is fixed to the value determined at T = 260 K ($\phi_{A(A')}$ = 16.6°, ϕ_B = -112.0° and ϕ_C = -92.5°), where the amplitude of the ψ-dependent Knight shift is close to the maximum at all sites, allowing an accurate determination of the phase. Supplementary Fig. 12, d-f, shows the T dependence of $K_j^{ab}(T)$ and $K_{j,\,\mathrm{aniso}}^{ab}(T)$ as well as $K_j(T,\psi)$ for a fixed ψ value on different sites. The three data excellently scale to each other over the entire T range at all sites (Supplementary Fig. 13), in good agreement with the observation at ambient pressure (*5*). This result indicates that the principal values of the tensor $\bar{\boldsymbol{\mathcal{A}}}_j$ show little T dependence, and, thus, the Knight shift directly probes the T dependence of the susceptibility: $K_j(T,\psi) = \bar{A}_j(\psi)\,\chi_s^j(T)$, where $\bar{A}_j(\psi)$ is a ψ-dependent hyperfine-coupling constant. Moreover, the ratio $K_j^{ab}/K_{j,\,\mathrm{aniso}}^{ab}$ at T = 300 K is ≈ 0.78, 0.73 and 0.84 for the non-equivalent site j = A(A'), B and C in the unit cell, respectively, which is to within ≈ 7% the same as what is reported at ambient pressure at T = 300 K (*5*). This clearly suggests that the anisotropy of $\bar{\boldsymbol{\mathcal{A}}}_j$ is little affected by P. All of these results, therefore, indicate that the coupling tensor in α-(BEDT-TTF)$_2$I$_3$ shows little T and P evolutions, which is consistent with earlier studies in similar BEDT-TTF-based compounds with stronger dimerization (the κ-type salts) (*6,17-20*). We thus employed the values of $\bar{\boldsymbol{\mathcal{A}}}_j$ reported at ambient pressure at T = 260 K (*5*) for calculating the ψ-dependent coupling constant $\bar{A}_j(\psi)$, converting $K_j(\psi)$ at P = 2.3 GPa to the site-spin susceptibility χ_s^j in terms of this $\bar{A}_j(\psi)$ at all measured temperatures (as shown in Fig. 2d).

Supplementary References

1. Katayama, S., Kobayashi, A. & Suzumura, Y. Electronic properties close to Dirac cone in two-dimensional organic conductor α-(BEDT-TTF)$_2$I$_3$. *Eur. Phys. J. B* **67**, 139-148 (2009).
2. Casula, M. *et al.*, Low-Energy Models for Correlated Materials: Bandwidth Renormalization from Coulombic Screening. *Phys. Rev. Lett.* **109**, 126408 (2012).
3. Isobe, H. & Nagaosa, N. Renormalization Effects on Quasi-Two-Dimensional Organic Conductor α-(BEDT-TTF)$_2$I$_3$. *J. Phys. Soc. Jpn.* **81**, 113704 (2012).
4. Kobayashi, A. & Suzumura, Y. Effects of Zero Line and Ferrimagnetic Fluctuation on Nuclear Magnetic Resonance for Dirac Electrons in Molecular Conductor α-(BEDT-TTF)$_2$I$_3$. *J. Phys. Soc. Jpn.* **82**, 054715 (2013).
5. Hirata, M., Ishikawa, K., Miyagawa, K., Kanonda, K. & Tamura, M. ^{13}C NMR study on the charge-disproportionated conducting state in the quasi-two-dimensional organic conductor α-(BEDT-TTF)$_2$I$_3$. *Phys. Rev. B* **84**, 125133 (2011).
6. Kawamoto, A., Miyagawa, K., Nakazawa, Y. & Kanoda, K. Electron correlation in the κ-phase family of BEDT-TTF compounds studied by ^{13}C NMR, where BEDT-TTF is bis(ethylenedithio)tetrathiafulvalene. *Phys. Rev. B* **52**, 15522 (1995).
7. Dóra, B. & Simon, F. Unusual hyperfine interaction of Dirac electrons and NMR spectroscopy in graphene. *Phys. Rev. Lett.* **102**, 197602 (2009).
8. Kondo, R., Kagoshima, S., Tajima, N. & Kato, R. Crystal and electronic structures of the quasi-two-dimensional organic conductor α-(BEDT-TTF)$_2$I$_3$ and its selenium analogue α-(BEDT-TSeF)$_2$I$_3$ under hydrostatic pressure at room temperature. *J. Phys. Soc. Jpn.* **78**, 114714 (2009).
9. Takano, Y., Hiraki, K., Takada, Y., Yamamoto, H. M. & Takahashi, T. Local Spin Susceptibility characteristic of zero-gap state of α-(BEDT-TTF)$_2$I$_3$ under pressure. *J. Phys. Soc. Jpn.* **79**, 104704 (2010).
10. Hirose, S. & Kawamoto, A. Local spin susceptibility in the zero-gap-semiconductor state of α-(BEDT-TTF)$_2$I$_3$ probed by ^{13}C NMR under pressure. *Phys. Rev. B* **82**, 115114 (2010).

11. Kakiuchi, T., Wakabayashi, Y., Sawa, H., Takahashi, T. & Nakamura, T. Charge Ordering in α-(BEDT-TTF)$_2$I$_3$ by Synchrotron X-ray Diffraction. *J. Phys. Soc. Jpn.* **76**, 113702 (2007).
12. Bender K. *et al.*, Synthesis, Structure and Physical Properties of a Two-Dimensional Organic Metal, Di[bis(ethylenedithiolo)tetrathiofulvalene]triiodide, (BEDT-TTF)$_2^+$I$_3^-$, *Mol. Cryst. Liq. Cryst.* **108**, 359-371 (1984).
13. Sugano, T., Saito, G. & Kinoshita, M. Conduction-electron-spin resonance in organic conductors: α and β phases of di[bis(ethylenedithiolo)tetrathiafulvalene]triiodide [(BEDT-TTF)$_2$I$_3$]. *Phys. Rev. B* **34**, 117-125 (1986).
14. Wojciechowski, R., Yamamoto, K., Yakushi, K., Inokuchi, M. & Kawamoto, A. High-pressure Raman study of the charge ordering in α-(BEDT-TTF)$_2$I$_3$. *Phys. Rev. B* **67**, 224105 (2003).
15. Schwenk, H. *et al.* α- and β-(BEDT-TTF)$_2$I$_3$ – two modifications with contrasting ground state properties: Insulator and volume superconductor. *Mol. Cryst. Liq. Cryst.* **119**, 329-335 (1985).
16. Tajima, N., Sugawara, S., Tamura, M., Nishio, Y. & Kajta, K. Electronic Phases in an Organic Conductor α-(BEDT-TTF)$_2$I$_3$: Ultra Narrow Gap Semiconductor, Superconductor, Metal, and Charge-Ordered Insulator. *J. Phys. Soc. Jpn.* **75**, 051010 (2006).
17. Mayaffre, H., Wzietek, P., Lenoir, C., Jérome, D. & Batail, P. ^{13}C NMR Study of a Quasi-Two-Dimensional Organic Superconductor κ-(ET)$_2$Cu[N(CN)$_2$]Br. *Eurphys. Lett.* **28**, 205-210 (1994).
18. De Soto, S. M. *et al.* ^{13}C NMR studies of the normal and superconducting states of the organic superconductor κ-(ET)$_2$Cu[N(CN)$_2$]Br. *Phys. Rev. B* **52**, 10364-10368 (1995).
19. Kawamoto, A., Miyagawa, K., Nakazawa, Y. & Kanoda, K. ^{13}C NMR Study of Layered Organic Superconductors Based on BEDT-TTF Molecules. *Phys. Rev. Lett.* **74**, 3455-3458 (1995).
20. Miyagawa, K., Kanoda, K. & Kawamoto, A. NMR Studies on Two-Dimensional Molecular Conductors and Superconductors: Mott Transition in κ-(BEDT-TTF)$_2$X. *Chem. Rev.* **104**, 5635-5653 (2004).
